# 15d-PGJ_2_-Loaded Solid Lipid Nanoparticles: Physicochemical Characterization and Evaluation of Pharmacological Effects on Inflammation

**DOI:** 10.1371/journal.pone.0161796

**Published:** 2016-08-30

**Authors:** Nathalie Ferreira Silva de Melo, Cristina Gomes de Macedo, Ricardo Bonfante, Henrique Ballassini Abdalla, Camila Morais Gonçalves da Silva, Tatiane Pasquoto, Renata de Lima, Leonardo Fernandes Fraceto, Juliana Trindade Clemente-Napimoga, Marcelo Henrique Napimoga

**Affiliations:** 1 Laboratory of Immunology and Molecular Biology, São Leopoldo Mandic Institute and Researcher Center, Campinas, Brazil; 2 Department of Environmental Engineering, São Paulo State University (UNESP), Sorocaba, Brazil; 3 Department of Physiological Sciences, Piracicaba Dental School, University of Campinas, Campinas, Brazil; 4 Department of Biochemistry, State University of Campinas (UNICAMP), Campinas, Brazil; 5 Department of Biotechnology, University of Sorocaba (UNISO), Sorocaba, Brazil; Indian Institute of Integrative Medicine CSIR, INDIA

## Abstract

15-deoxy-Δ^12,14^-prostaglandin J_2_ (15d-PGJ_2_), a peroxisome proliferator-activated receptor-γ (PPAR-γ) agonist, has physiological properties including pronounced anti-inflammatory activity, though it binds strongly to serum albumin. The use of solid lipid nanoparticles (SLN) can improve therapeutic properties increasing drug efficiency and availability. 15d-PGJ_2_-SLN was therefore developed and investigated in terms of its immunomodulatory potential. 15d-PGJ_2_-SLN and unloaded SLN were physicochemically characterized and experiments *in vivo* were performed. Animals were pretreated with 15d-PGJ_2_-SLN at concentrations of 3, 10 or 30 μg·kg^-1^ before inflammatory stimulus with carrageenan (Cg), lipopolysaccharide (LPS) or mBSA (immune response). Interleukins (IL-1β, IL-10 and IL-17) levels were also evaluated in exudates. The 15d-PGJ_2_-SLN system showed good colloidal parameters and encapsulation efficiency of 96%. The results showed that the formulation was stable for up to 120 days with low hemolytic effects. The 15d-PGJ_2_-SLN formulation was able to reduce neutrophil migration in three inflammation models tested using low concentrations of 15d-PGJ_2_. Additionally, 15d-PGJ_2_-SLN increased IL-10 levels and reduced IL-1β as well as IL-17 in peritoneal fluid. The new 15d-PGJ_2_-SLN formulation highlights perspectives of a potent anti-inflammatory system using low concentrations of 15d-PGJ_2_.

## Introduction

Drug delivery systems have been developed to prolong and improve drug action while reducing toxicity, thus overcoming common problems such as poor solubility in water [1,2].

Solid lipid nanoparticles (SLN), submicron lipid carriers sized between 50 and 1000 nm, are composed of biocompatible materials able to incorporate mainly lipophilic drugs. SLN are constituted by an external phase (an emulsifier and water) and an inner layer composed of lipid matrix, where the drug is dispersed [3–5]. Such nanocarriers feature low toxicity and cause no irritation to tissues, hence the growing interest in their use in the treatment of inflammatory diseases [6].

Inflammation is an organic response that precedes tissue injury or infection. This physiological process involves a coordinated action between the immune system and the damaged tissue, which stimulates infiltration and subsequent activation of inflammatory cells with release of cytokines and other mediators [7]. In such scenario, 15-deoxy-Δ^12, 14^-prostaglandin J_2_ (15d-PGJ_2_) stands out as a potential anti-inflammatory molecule. This kind of prostaglandin is a PPAR-γ (peroxisome proliferator-activated receptor-γ) agonist and it is derived from the cyclooxygenase pathway, participating in the resolution phase of acute inflammation [8–10].

Studies have shown that PPAR-γ agonists are being used in inflammatory disorders because they are able to regulate the immune response [9–14]. In a previous study, it was demonstrated that 1 mg·kg^-1^ of plain 15d-PGJ_2_ is required to reduce neutrophil migration to an inflammation site [12] due to the high affinity of such molecule to serum proteins [8]. In order to increase the bioaviability and improve the pharmacological properties of 15d-PGJ_2_, our group demonstrated that PLGA-encapsulated 15d-PGJ_2_ achieved the same therapeutic effect as free 15d- PGJ_2_ in several inflammation models at a concentration 33 times lower than the latter [15]. Encapsulated 15d-PGJ_2_ was also able to inhibit bone loss in periodontal disease secondary to reduced gingival inflammation [16]. Such findings highlight the promising applications of 15d- PGJ_2_ encapsulated in nanoparticles.

The new formulation addressed in this study has advantages over other colloidal nanocarriers such as polymeric nanoparticles. Besides being made of physiologically well-tolerated materials, SLN have been approved for human use based on their low toxicity, reportedly 10 to 100 times lower than polymeric nanoparticles. SLN also have good stability and wide range of administration routes, including parenteral [3,5]. SLN are produced from lipids that are solid at room temperature such as mono-, di- or triglycerides, fatty acids or waxes. Such lipids are stabilized by one or a mixture of emulsifiers to prevent nanoparticle agglomeration. Most such materials are currently used in pharmaceutical or cosmetic formulations, which highlights their low toxicity [17].

SLN have shown to be better tolerated *in vivo* than polymeric nanoparticles, since the polymers used in the latter may carry an intrinsic element of cytotoxicity [18,19]. Moreover, the composition of the formulation used in this study, namely tripalmitin as lipid matrix and PVA as emulsifier, has been shown to be suitable as a sustained delivery system of bioactive molecules due to their good physicochemical characteristics such as size, zeta potential, high encapsulation efficiency, etc [20,21].

There have only been a few reports on the association of 15d-PGJ_2_ to lipid systems [22], though none amounted to a study *in vivo* on the anti-inflammatory activity of such systems. The purpose of this study was therefore to address the physicochemical properties and the potential anti-inflammatory activity of the novel 15d-PGJ_2_-SLN formulation *in vivo*.

## Materials and Methods

### Preparation of 15d-PGJ_2_-Loaded SLN

The nanoparticles were prepared via the emulsification/solvent evaporation method described elsewhere [21,23], with some modifications. Firstly, the organic phase composed by the lipid glyceryl tripalmitate (150 mg) and 15d-PGJ_2_ (100 μg) was dissolved in chloroform (5 mL). The aqueous phase was composed by polyvinyl alcohol (1%, w/v) and deionized water (30 mL). The organic phase was added to the aqueous phase and this mixture was sonicated for 5 min at 40 W on a probe sonicator yielding an emulsion, which in turn was mixed on an Ultra Turrax homogenizer at 18,000 rpm for 7 min. The organic solvent was eliminated under low pressure using a rotating evaporator and the final volume was 16 mL of 15d-PGJ_2_ at a concentration of 6.25 μg.mL^-1^. A control formulation was also prepared without 15d-PGJ_2._

### Encapsulation Efficiency of 15d-PGJ_2_

The amount of 15d-PGJ_2_ encapsulated by the SLN system was determined by ultrafiltration/centrifugation using Amicon^®^ ultrafiltration devices (10 kDa MWCO; Millipore^®^). 15d-PGJ_2_-SLN suspensions were centrifuged and the filtrate was analyzed using high performance liquid chromatography (HPLC). 15d-PGJ_2_ was quantified in a Varian ProStar equipment (Agilent^®^ Technologies), PS210 isocratic pump and a UV-Vis detector operating at 205 nm. A Gemini^®^ C_18_ column NX 5μ C_18_ 110 Å, 150 x 4.6 mm (Phenomenex^®^) was used for the mobile phase of sodium phosphate monobasic solution (pH 3.5; 0.01M) and acetonitrile (58:42, v/v) at 1 mL.min^-1^. The sample injection volume was 100 μL [15]. Encapsulation efficiency was then determined from the difference between 15d-PGJ_2_ concentration measured in the ultrafiltrate and its total concentration (100%) in the nanoparticle suspension.

### Size, Polydispersion, Zeta Potential and pH Measurements

The hydrodynamic diameter and polydispersion (PI) of nanoparticles were determinated using dynamic light scattering and the zeta potential was evaluated by microeletrophoresis. A ZetaSizer Nano ZS 90 analyzer (Malvern^®^ Instruments) was used to perform the measurements (25°C, with an angle of 90°) with a dilution factor of 100 times. The pH values of the suspensions were evaluated using a calibrated pHmeter (Tecnal^®^, Brazil). The results were shown as the means of five measures (mean ± SD). The physicochemical stability of the nanoparticles was evaluated as a function of time, analyzing the suspension over 120 days [15,21,24].

### Nanoparticle Tracking Analysis (NTA)

SLN size distribution was investigated through NTA. The analyses were carried out on a NanoSight LM 10 instrument system, green laser beam (532 nm) and sCMOS camera, all controlled via dedicated NanoSight v.2.3 software (Malvern^®^ Instruments, UK). Samples were diluted 10,000 times and analyzed in triplicate injecting the sample (1 mL) into the cell. Five individual videos of Brownian motion were recorded for each replicate at two thousand particles per replicate. Finally, the result achieved was the concentration of the particles as a function of size distribution [20,21].

### Determination of Nanoparticle Morphology

Nanoparticle morphology was determined using transmission electron microscopy (TEM). SLN suspensions with or without the drug were diluted and applied to copper grids (200-mesh) coated with carbon film. The samples were dried at room temperature and were contrasted using uranyl acetate (2%) and analyzed using a Zeiss LEO 906 microscope operating at 80 kV, preserving the nanoparticle morphology [21,25].

### *In Vitro* Release Assay

The release profile of encapsulated 15d-PGJ_2_ in SLN was evaluated employing a donor-acceptor compartments system, with an interposed cellulose membrane (MWCO 1 kDa). The system was maintained under constant magnetic stirring and *sink* conditions. Samples were applied to the surface of the donor compartment. From the acceptor compartment, aliquots of 1 mL were collected over 6 hours. The concentration of the drug released was determined by HPLC in the acceptor compartment. The experiment was carried out in triplicate [16,24].

The release mechanism of SLN-encapsulated 15d-PGJ_2_ was evaluated using the Baker-Lonsdale theoretical model. This is based on the Higuchi model and is intended at elucidating drug release phenomena from spherical devices, such as micro and nanoparticles [26].

### Evaluation of Cellular Viability

Cell viability experiment was established using the MTT reduction test. The experiment was performed on Balb-c 3T3 fibroblasts cultured in DMEM (supplemented with 10% fetal bovine serum, 1% penincilin and streptomycin sulfate). Briefly, cells were seeded into culture plates and incubated for 48 h. They were then exposed to free 15d-PGJ_2_ and SLN suspension (with and without 15d-PGJ_2_) at drug concentrations ranging from 0.06 to 2.2 μg.mL^-1^ for 24 hours. The cells were then incubated with MTT for 2 h at 37°C and purple formazan was quantified using a plate reader at 570 nm. Cell viability was calculated as absorbance of converted dye [20,21,24].

### Animals

Male Balb/c mice (20–25 g) were used in this investigation. The animals were maintained in a temperature-controlled room (12:12 h light–dark cycle) and provided water and food *ad libitum*. This study was approved by the Ethics Committee on Animal Research of the University of Campinas (registration number 3623-1/2015) and all animals were manipulated in accordance with the *Guiding Principles for the Care and Use of Animals*.

### Antigen-Induced Peritonitis

The immunization procedure occurred as previously described [15]. On day one, the animals were exposed to the antigen via a subcutaneous (s.c.) injection of 100 μL of saline, 100 μL of complete Freund’s adjuvant and 500 μg of methylated bovine serum albumin (mBSA) antigen. The animals were boosted on days 7 and 14 with mBSA dissolved in incomplete Freund’s adjuvant. Non-immunized (NI) animals received similar treatment, but without the antigen. All animals were treated with 15d-PGJ_2_-SLN at 3, 10 or 30 μg·kg^-1^ or empty SLN and challenged with either mBSA (30 μg/cavity, intraperitoneal (i.p.)) or saline control on day 21. Four hours after the mBSA challenge, the animals were sacrificed by isoflurane inhalation and the peritoneal cavity was washed with 3 mL of phosphate buffered saline (PBS) containing 1 mM ethylenediamine tetraacetic acid (EDTA) and the exudate was recovered [15,27].

### Carrageenan-Induced Peritonitis and LPS-Induced Neutrophil Migration

Thirty minutes prior to the challenge, the animals were pretreated subcutaneously with 200 μL of saline or 15d-PGJ_2_-SLN (3, 10 or 30 μg·kg^-1^). Inflammation was caused by i.p. injection of carrageenan (Cg at 500 μg/cavity in 200 μL). A control group with empty SLN was included aiming to verify whether the vehicle alone could affect neutrophil migration. Two hundred microliters of vehicle were injected s.c. and after 30 minutes the animals were challenged with saline or Cg (i.p.). As previously described, 4 hours after the Cg challenge, the animals were sacrificed and the peritoneal cavity was washed with PBS containing 1 mM EDTA and the peritoneal fluid recovered [15].

For LPS-induced neutrophil migration, the animals were pretreated s.c. with saline (200 μL), 15d-PGJ_2_-SLN (3, 10 or 30 μg·kg^-1^) or empty SLN. Thirty minutes later, LPS solution was injected (100 ng/cavity, i.p.). Four hours after the LPS challenge, all animals underwent the same procedure described for antigen-induced and Cg-induced peritonitis [15].

### Cell Counts and Cytokine Measurements

A Neubauer chamber was used for total cell counts, with samples diluted in Turk’s solution. Differential cell counts were determined from cytocentrifuge monolayers stained with Wright-Giemsa (100 counted cells). The results were expressed as the number of neutrophils per cavity (means ± SD) [15].

IL-1β, IL-10 and IL-17 levels in the peritoneal fluid were determined using commercial kits of enzyme-linked immunosorbent assay (ELISA) (R&D Systems, USA) at optical density of 490 nm. From the standard curves, the results were expressed as pg·mL^-1^ of cytokine.

### Hemolytic Assay

The hemolytic effect provoked by nanoparticle suspensions was evaluated using mouse erythrocytes (0.15% hematocrit). Hemolysis percentage was determined by the quantity of hemoglobin released from red blood cells. The samples were incubated with 15d-PGJ_2_-SLN suspensions at concentrations ranging from 6.25 to 125 ng.mL^-1^ of the drug. Empty SLN were evaluated at the same nanoparticle concentration (~1.60 X 10^13^ nanoparticles/mL) tested for loaded nanoparticles. Erythrocytes were incubated at 37°C for 15 min and centrifuged at 1500x g for 3 min. Hemoglobin in the supernatant was detected at 412 nm wavelength. The data were expressed as hemolysis percentage [15,28].

### Statistical Analysis

Five animals were used per group for the experiments *in vivo*. Data were reported as means ± SD. Different treatments were compared using ANOVA and Bonferroni’s *t*-test for unpaired values. For the cytotoxic assays, data were analyzed using ANOVA with Tukey’s *post hoc* test. Statistical significance was set at *P<*0.05.

### Results

Both SLN suspensions (empty and 15d-PGJ_2_ loaded) were prepared and colloidal parameters evaluated. The nanoparticles were measured for hydrodynamic diameter, polydispersion, zeta potential and morphology. The rationale behind developing lipid nanoparticles includes the relatively low costs of the raw materials and production as well as the excellent physicochemical stability inherent of such preparations. The colloidal parameters are described in Table 1.

**Table 1:** Colloidal parameters of both SLN and 15d-PGJ_2_-SLN suspensions. Values are expressed as means ±standard deviations.

Parameters	SLN (1^st^ day)	15d-PGJ_2_-SLN (1^st^ day)	SLN (120^th^ day)	15d-PGJ_2_-SLN (120^th^ day)
**Mean diameter (nm)**	252.3 ± 8.7	283.6 ± 8.1	260.2 ± 4.9	289.5 ± 2.3
**Polydispersion**	0.105 ± 0.013	0.109 ± 0.048	0.147 ± 0.024	0.113 ± 0.017
**Zeta potential (mV)**	-23.6 ± 0.3	-24.8 ± 0.7	-21.9± 1.1	-17.8± 0.3
**pH**	4.43 ± 0.06	4.41 ± 0.04	5.11 ± 0.02	5.22 ± 0.01
**Encapsulation efficiency (%)**	-	96.8 ± 1.7	-	95.1 ± 0.8

The results showed that the parameters evaluated for the nano suspensions were similar to those described for colloidal suspensions and that such parameters were not affected by encapsulation of the active ingredient [21]. The polydispersion index values were below 0.2 for both suspensions, indicating a narrow distribution of particle diameter; a negative zeta potential was found for both nano suspensions. Encapsulation efficiency was up to 95%. This high value is a combination of the lipophilicity and affinity of the drug for the lipid core.

The physicochemical stability of particles is important data to define suitability of formulations. The parameters size, polydispersion, zeta potential, pH and encapsulation efficiency were monitored over 120 days of storage in ambar glass flasks. The size of nanoparticles in both suspensions analyzed had such little variation that it may be regarded as constant throughout the period of 120 days, indicating no aggregation of particles. The remaining parameters followed suit, indicating suitable colloidal stability of the systems [20,21,29].

**Fig 1:**
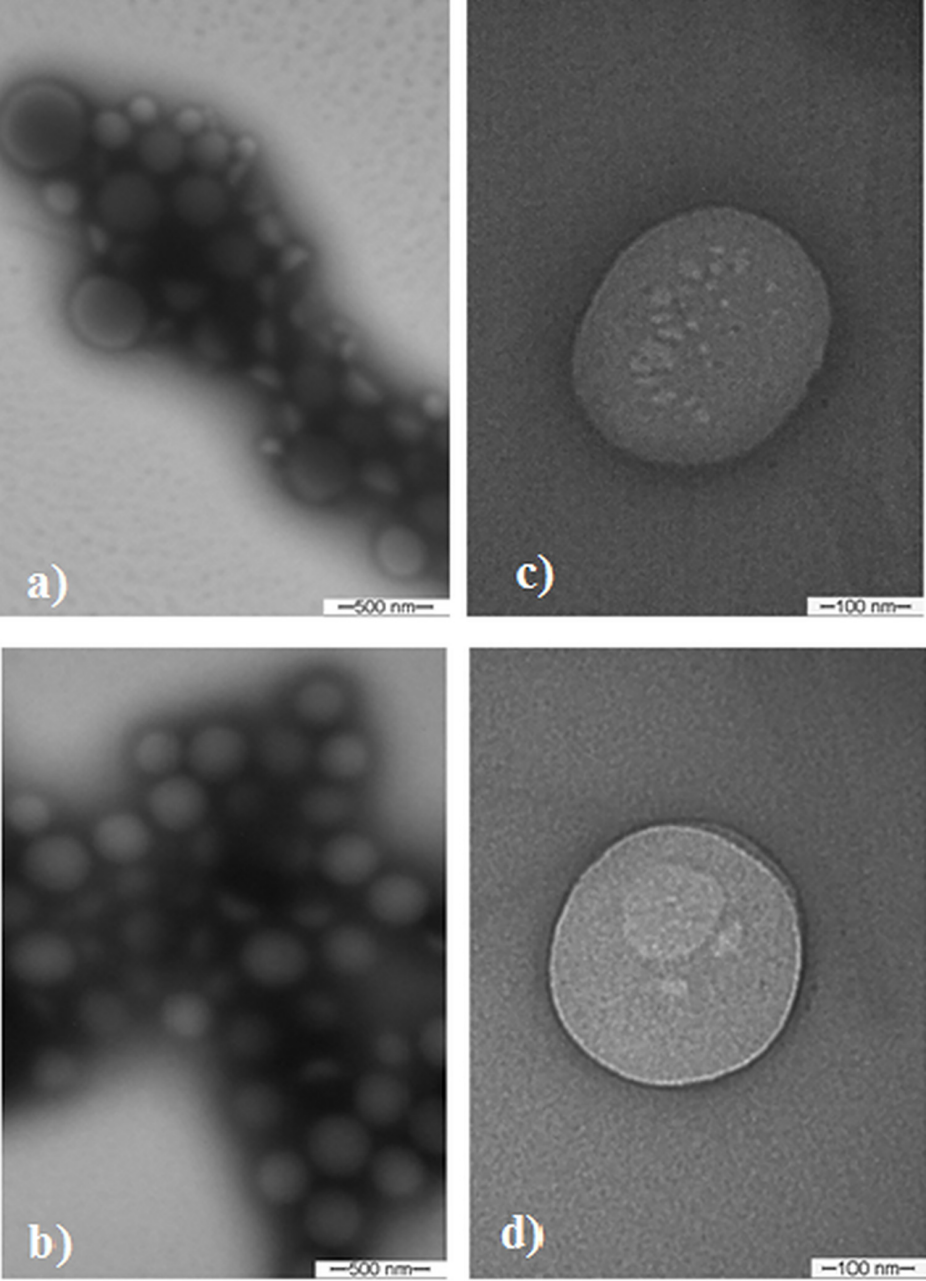
Micrographs obtained through transmission electron microscopy. (a-b) SLN and 15d-PGJ_2_-SLN at 77.500x magnification; (c-d) SLN and 15d-PGJ_2_-SLN at magnification 215.000x magnification. The bars indicate image scales.

TEM micrographs obtained for the SLN and 15d-PGJ_2_-SLN preparations are shown in Fig 1. The nanoparticles presented a spherical shape with diameters in the range of 200–300 nm (Fig 1A–1D). The presence of agglomerates was a technical artifact that occurred secondary to sample drying during processing for TEM analysis. Particle sizes were consistent with both the polydispersion index and the mean diameter values obtained with the DLS technique [21,29].

**Fig 2:**
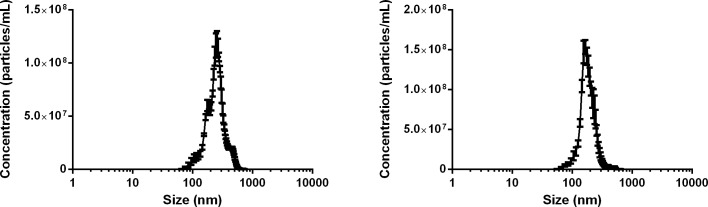
Nanoparticle concentration as a function of particle size (nm). (a) SLN and (b) 15d-PGJ_2_-SLN performed at 25°C (n = 5).

The NTA technique was used to characterize the SLN system. The results of nanoparticle concentration are shown in Fig 2. For empty SLN, the concentration was 1.90 (± 0.35) × 10^13^ particles per mL with average diameter of 274.6 ± 93.5 nm. For 15d-PGJ_2_–SLN the concentration was 1.60 (± 0.69) × 10^13^ particles per mL with average diameter of 194.1 ± 61.9 nm. Both DLS and NTA provided similar range of diameter values and particle concentration, with monomodal size distributions. This was an indication of homogeneity of the system, which is desirable in nanoparticulated formulations, as it reduces interferences with drug-release by increasing stability.

**Fig 3:**
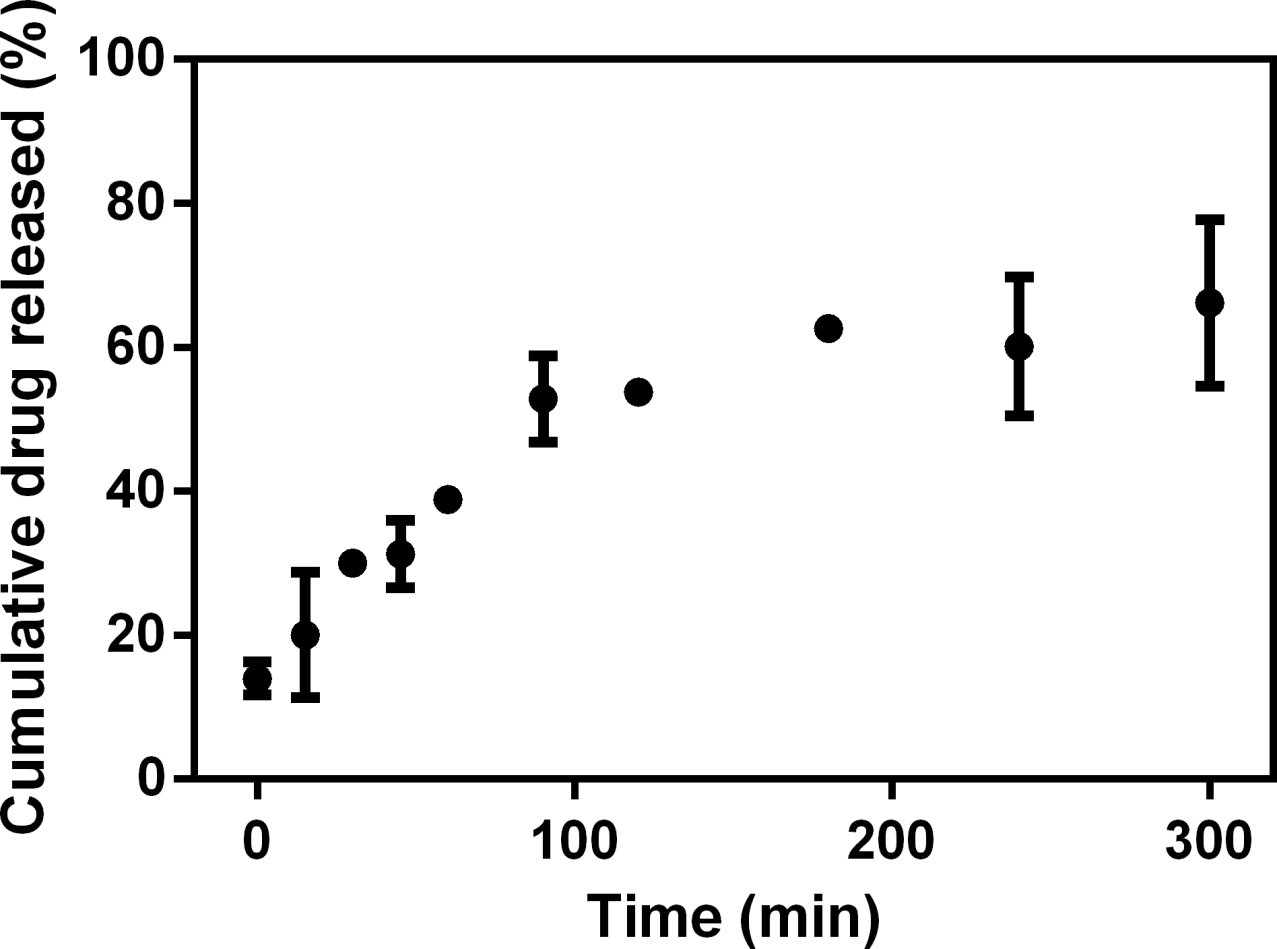
Cumulative release profile of 15d-PGJ_2_–loaded SLN *in vitro* in aqueous solution at 25°C (n = 3).

The release profile of 15d-PGJ_2_ from SLN suspension was investigated (Fig 3). In this model, only free molecules of the drug were able to transpose the membrane, while the nanoparticles were retained, which enabled the evaluation of the interaction between drug and nanoparticles. 15d-PGJ_2_ was quantified by HPLC in aliquots collected from the acceptor.

Fig 3 illustrates the release profile of 15d-PGJ_2_ from the SLN system. The time taken for 50% release (t_50%_) was around 100 min. Evaluation of the release mechanism of 15d-PGJ_2_ from the SLN systems was performed using the Baker-Lonsdale´s equation. Linear regression resulted in correlation coefficient (*r*) values of 0.982, and release constant (*k*) values of 0.322 x 10^−2^ (± 0.001) min^-1^. Compared with the release profile of 15d-PGJ_2_ from PLGA nanocapsules, the best fit was achieved using the Higuchi´s model (*r* = 0.972; *k* = 0.048 min^-1/2^) and the main release mechanism involved is based on the Fick's Law of diffusion [16]. In this study, we achieved compatibility with the Baker–Lonsdale model indicating that the mechanism involved is also diffusion. With respect to *k* values, the release profile obtained from 15d-PGJ_2_-SLN was lower than that from PLGA nanocapsules. This could be explained by the superior interaction between 15d-PGJ_2_ and the tripalmitin used in the production of SLN [30], also demonstrated via the encapsulation efficiency values (>95% for SLN and 77% for PLGA nanocapsules). This high interaction caused slower release, showing that the SLN system was more suitable for the 15d-PGJ_2_ molecule.

Cell viability after exposure to SLN suspensions was assessed using the MTT reduction test. The experiments were performed in 3T3 fibroblasts incubated with free 15d-PGJ_2_ and encapsulated 15d-PGJ_2_ (0.06–2.2μg.mL^-1^). Controls using empty nanoparticles were also tested at the same nanoparticle concentration (~ 1.60 x 10^13^ nanoparticles/mL).

**Fig 4:**
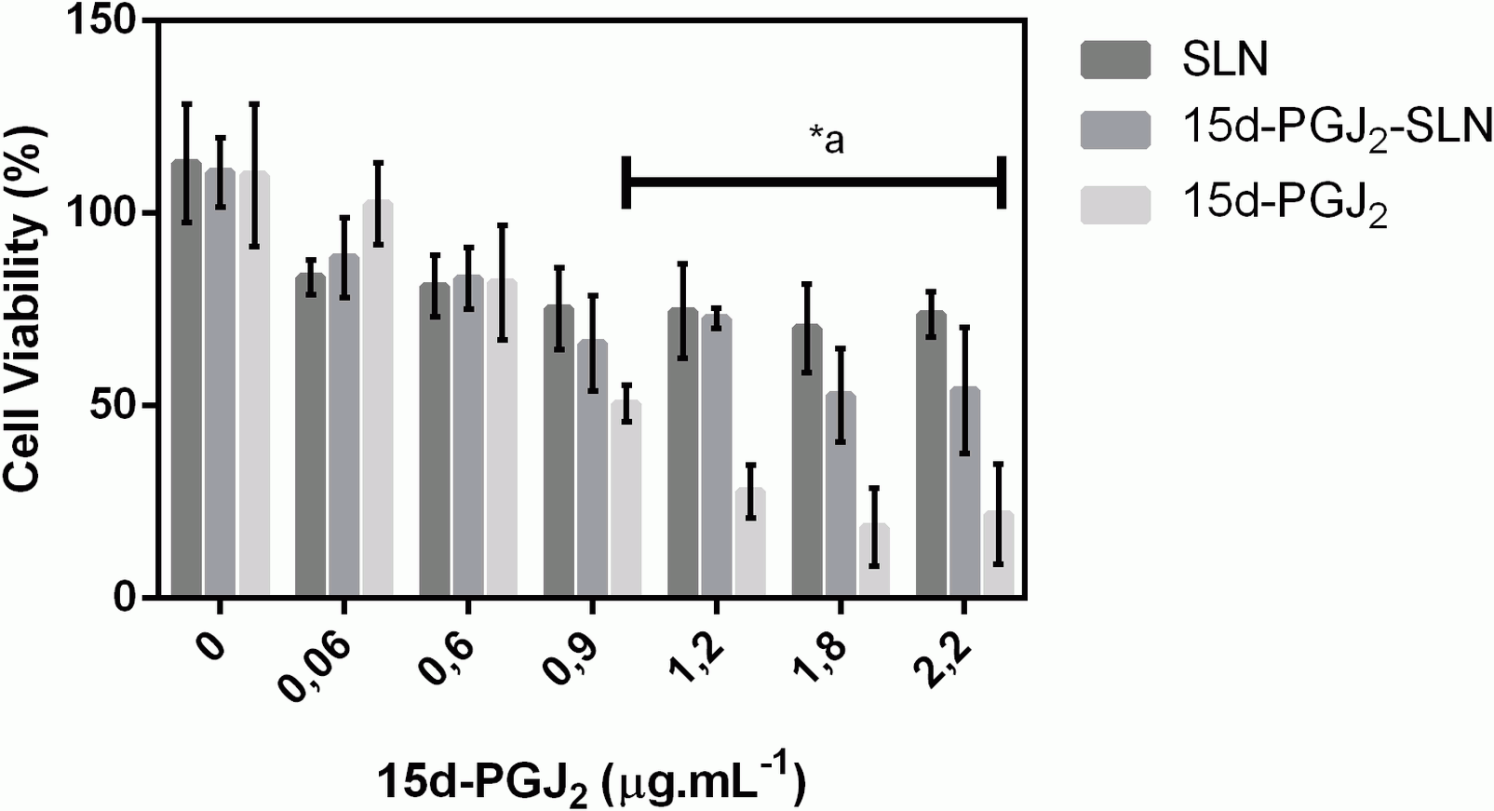
Viability of Balb-c 3T3 cells using the MTT assay after exposure to free 15d-PGJ_2_ and both nanosuspensions (n = 12). Data are represented as percentage of viable cells. (*a) 15d-PGJ_2_-SLN versus 15d-PGJ_2_ (ANOVA followed by Tukey test; *P* < 0.05).

It is known that some ionizable groups present in polymers, lipids and other components of the suspensions could be somewhat toxic due to their interaction with cell membranes [19,31]. The results (Fig 4) showed that SLN decreased cell viability to 70% at the highest concentration tested. Exposure to free 15d-PGJ_2_ reduced cell viability to 30%, mainly at higher concentrations, while exposure to the 15d-PGJ_2_-SLN formulation significantly increased cell viability at prostaglandin concentrations ranging from 0.9 to 2.2 μg.mL^-1^ (*P*< 0.05).

**Fig 5:**
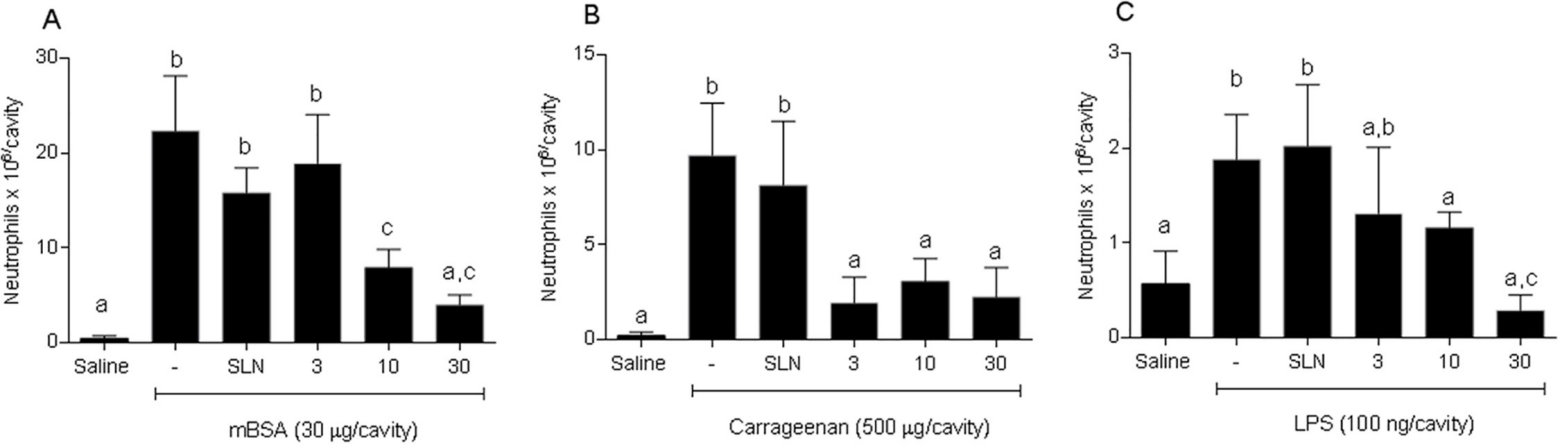
Anti-inflammatory effect of SLN and 15d-PGJ_2_-SLN assessed in different inflammatory models. The animals were tested using saline or 15d-PGJ_2_-SLN (3, 10 or 30 μg·kg^−1^) prior to challenging them with mBSA (30 μg/cavity) (A), Cg (500 μg/cavity) (B) or LPS (100 ng/cavity) (C). Neutrophil migration was evaluated 4h later. Results are expressed as mean values (±SD) of 5 animals per group. Different letters indicate statistical significance between groups (ANOVA followed by Bonferroni's *t*-test; *P* < 0.05).

I.p. administration of mBSA in immunized animals significantly increased neutrophil migration in the peritoneal cavity compared to saline or to mBSA in NI animals. Pretreatment with encapsulated 15d-PGJ_2_ at three concentrations (3, 10 or 30 μg·kg^-1^) was able to inhibit neutrophil migration (3, 10 or 30 μg·kg^-1^) (Fig 5A). Cg and LPS injections significantly increased neutrophil migration *in vivo* compared to saline. Similarly to mBSA, administration of the 15d-PGJ_2_-SLN formulation sharply decreased neutrophil migration induced by either stimuli (Fig 5B and 5C) (*P* < 0.05). Empty SLN were also tested in three models to verify whether the nanoparticles were able to alter immune responses. The results indicate no significant change in neutrophil migration (*P* > 0.05). Similar findings were previously reported for 15d-PGJ_2_-loaded polymeric nanocapsules [15].

**Fig 6:**
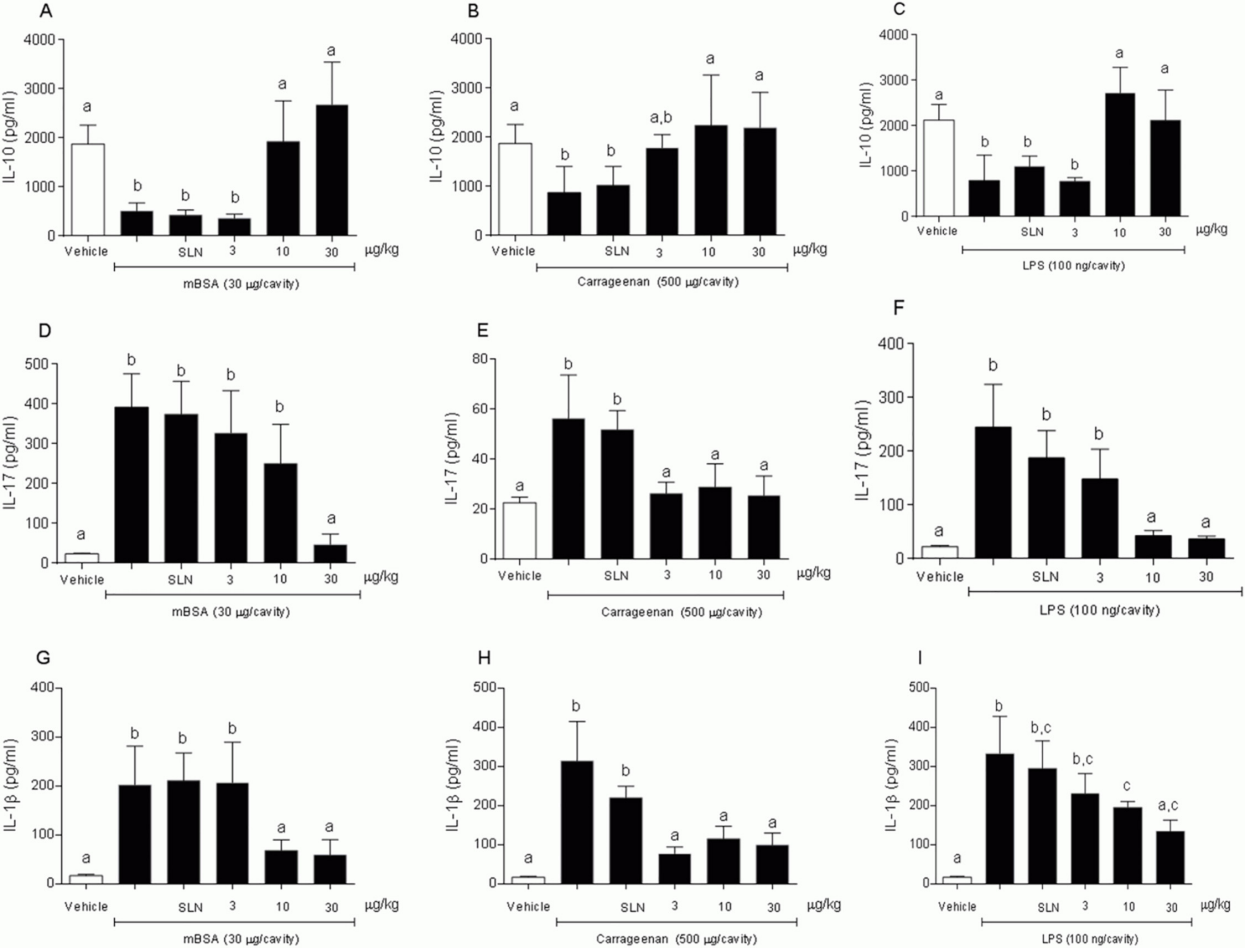
Influence of 15d-PGJ_2_-SLN (3, 10 or 30 mg·kg^-1^) on IL-10, IL 17 and IL-1β levels in exudate. (A, D, G) mBSA (30 μg/cavity), (B, E, H) Cg (500 μg/cavity), (C, F, I) LPS (100 ng/cavity). Results are expressed as mean values (±SD) of 5 animals per group. Different letters indicate statistical significance between groups (ANOVA followed by Bonferroni's *t*-test; *P* < 0.05).

IL-10 release induced by 15d-PGJ_2_-SLN was investigated in order to evaluate a possible regulatory effect on neutrophil migration. A dose-dependent increase in IL-10 levels was observed in the peritoneal fluid of mice tested with 15d-PGJ_2_-SLN and challenged with mBSA or Cg or LPS. For mice tested with saline or SLN, no increase in IL-10 levels were observed in either of the three inflammation models (Fig 6A–6C).

Release of pro-inflammatory cytokines such as IL- 1β (Fig 6D–6F) and IL -17 (Fig 6G–6I) was also evaluated since reduction in neutrophil migration could accompany a diminished release of chemotactic agents. A dose-dependent decrease in IL-1β and IL-17 levels was observed in the peritoneal exudate of mice tested with 15d-PGJ_2_-SLN and challenged with mBSA, Cg or LPS, whereas the mice tested with saline or SLN alone showed no decrease in cytokine levels for the inflammation models assessed.

**Fig 7:**
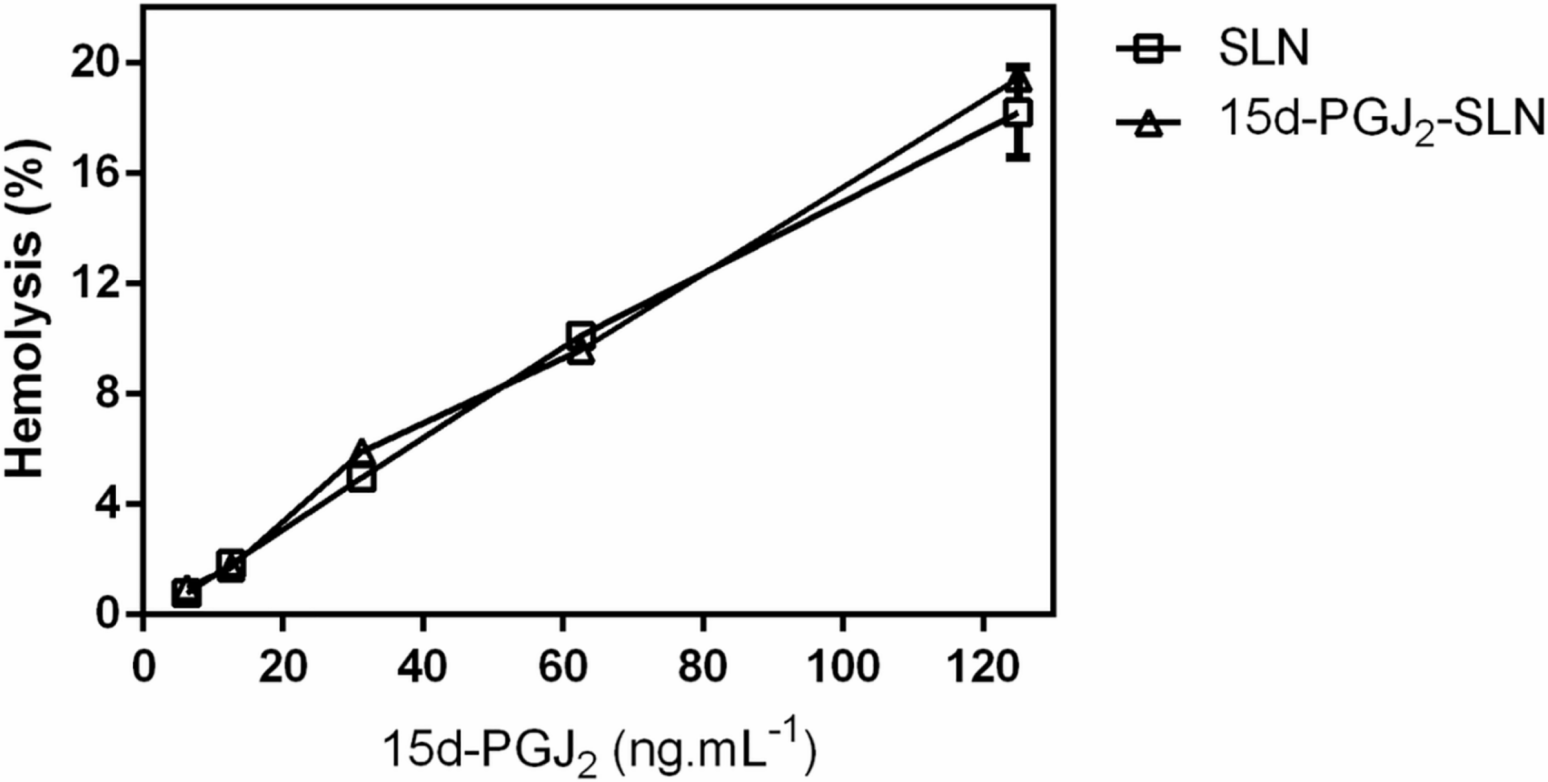
Hemolysis assay employing 0.15% hematocrit, pH 7.4 at 37°C for SLN and 15d-PGJ_2_-SLN suspensions (n = 6).

The hemolytic effect of both loaded and unloaded SLN were investigated using red blood cells from mice (Fig 7). Exposure of cells to both formulations demonstrated low toxicity, as illustrated by the hemolysis values obtained even with the highest concentration tested (125 ng.mL^-1^) (~18% for SLN and ~19% for 15d-PGJ_2_-SLN suspensions). Blood toxicity of the empty PLGA nanocapsules and those containing 15d-PGJ_2_ was tested employing a hemolysis assay. The results revealed a low toxicity of such formulations even at the highest concentration (125 ng.mL^-1^), with 40% and 35% hemolysis reported for the empty and loaded nanocapsules, respectively [15], while for the SLN suspensions (empty and loaded nanoparticles), 18.2% and 19.4%, respectively. The hemolytic effect was therefore lower for the SLN than for the PLGA nanocapsules, consequently blood-compatibility was higher for the former, though the difference between the formulations was not statistically significant.

## Discussion

Nanoencapsulation has gained great interest in the pharmaceutical field. Among the nanoparticles used to encapsulate drugs, SLN can be highlighted due to its high stability, small size, protection of labile drugs as well a change in their release profile [32]. The SLN systems presented good colloidal stability, as well as high affinity to the lipid matrix. The SLN system was able to modify drug release profile and to maintain cell viability compared to the free drug. 15d-PGJ_2_ encapsulation reduced neutrophil migration in 3 different inflammatory models due in part to a decrease in IL-1β and IL-17 levels as well an increase in IL-10.

The parameters mean diameter and polydispersion index remained practically constant, with no aggregates, demonstrating the high stability of such systems over the study period. Other critical parameter for colloidal stability is zeta potential. This parameter reflects nanoparticles surface charge. Some surfactants used in this formulation, such as PVA, however, have a steric stabilization mechanism. In this case, the surface charge measured cannot be used as the main stability parameter [1,20]. Nonetheless, the zeta potential values were not affected by encapsulation of the drug, which indicates that the drug is mostly contained within the nanoparticles. Such findings demonstrate that the physicochemical stability of the SLN suspension is highly suitable for 15d-PGJ_2_ encapsulation. Similar results have been reported for other SLN suspensions [20,21,29].

As demonstrated, 15d-PGJ_2_ encapsulation into SLN was highly efficient (>95%), confirming the affinity of the drug to the lipid phase of the SLN. It has previously been reported that the encapsulation efficiency of PLGA nanocapsules was approximately 77% for 15d-PGJ_2_ [15]_._ SLN has therefore been proved more suitable to encapsulate 15d-PGJ_2_, due probably to the greater affinity or higher solubility of such drug in the lipids used in this study.

The release profile of the 15d-PGJ_2_-SLN formulation was evaluated using a two-compartment model. Because of the low solubility of 15d-PGJ_2_ in water, it was not possible to investigate the release profile of the free drug. Comparing with a previous report, the system 15d-PGJ_2_-SLN presented a lower release profile than the drug encapsulated in polymeric nanocapsules [16], which is desirable and indicates a technical advantage of the latter over the former.

The cell viability pattern observed for unloaded SLN could be attributed to exposure of the cells to residual PVA present in the formulation or even to the influence of the nanoparticle charge. A study has shown that tripalmitin SLN reduced cell viability to 30% and this could be attributed to the formation of aggregates and changes in nanoparticle structure leading to reduced of cell viability [33]. Our experimental data showed that high doses (0.9–2.2 μg.mL^-1^) of free 15d-PGJ_2_ resulted in lower cell viability than the 15d-PGJ_2_-loaded SLN (*P*<0.05). Such protective an effect may be explained by a reduced availability of the drug secondary to a high encapsulation efficiency and modified release profile obtained with the nanoparticles [19]. On the other hand, taking into account the *in vivo* data, the dose of 10 μg.kg^-1^ was effective in all 3 inflammatory models. This concentration normalized by body size means that the final concentration was approximately 0.3 μg. From both the *in vitro* and *in vivo* findings, one may conclude that the effective dose used did not induce significant side effects. Thus, the results confirmed that 15d-PGJ_2_ encapsulation into SLN resulted in reduced drug availability, thereby favoring a higher percentage of viable cells.

The hemolytic effects of SLN suspensions were evaluated in mouse erythrocytes in order to investigate blood compatibility of such formulations. Solid lipid nanoparticles are regarded as biocompatible and biodegradable systems. This may be related to their physicochemical properties, lipid composition, etc [34]. The results herein demonstrated that both loaded and unloaded formulations caused low hemolysis (below 20%) indicating biocompatibility. The hemolytic effect observed was dose-dependent and could also be attributed to exposure to residual PVA. Despite both formulations being biocompatible, SLN was superior at delivering 15d-PGJ_2_ in biological systems.

Comparing the present findings to those obtained with PLGA nanocapsules published previously [15], it is evident that SLN performed better than the polymeric nanocapsules.

It has been described that high doses of exogenous 15d-PGJ_2_ are needed to exert pharmacological effects [11,12] and encapsulation of the drug into polymeric nanocapsules was able to protect the molecule and promote anti-inflammatory effects at low doses [15,16,35]. In this investigation, our results showed that the 15d-PGJ_2_-SLN system had an anti-inflammatory activity. In all three inflammatory models tested, 15d-PGJ_2_-SLN inhibited leukocyte migration to an inflammatory site and, most importantly, 15d-PGJ_2_-SLN at 10 μg.kg^-1^ was able to significantly decrease neutrophil migration and pro-inflammatory cytokine levels (IL-1β and IL-17) as well as to significantly raise IL-10 levels. Compared to previous data using free 15d-PGJ_2_ (1000 μg.kg^-1^) [12], the current formulation (15d-PGJ_2_-SLN) lowered the dose needed to obtain an anti-inflammatory effect by 100 times. Additionally, compared to the previous formulation tested (PLGA nanocapsules) [15], for which the effective dose was 30 μg.kg^-1^, this new nanocarrier managed to further reduce the dose 3-fold, demonstrating that the SLN system was the most effective at releasing the drug at the site of inflammation. The effect of free 15d-PGJ_2_ was not evaluated in this study because it has previously been established that the free drug at such low concentration does not exert an anti-inflammatory effect [15].

The advantages of this new carrier are higher stability, lower preparation costs and higher efficiency at releasing 15d-PGJ_2_ in three animal inflammation models compared to PLGA nanocapsules, highlighting its potential as an alternative delivery method for 15d-PGJ_2_. The results obtained herein open new perspectives and represent a great step towards the treatment of inflammatory diseases using a sustained 15d-PGJ_2_ releasing method from nanoparticles.
